# Self-harm as a form of resistance in the juvenile detention center Jugendhaus Halle in the 1980s

**DOI:** 10.1186/s13034-025-00994-2

**Published:** 2025-11-15

**Authors:** Oxana Kosenko, Florian Steger

**Affiliations:** Institute of the History, Philosophy and Ethics of Medicine at Ulm University, D-89081 Ulm, Germany

**Keywords:** Self-harm, Suicide, Juvenile prisons, Ethics, Forensics, Germany

## Abstract

**Background:**

Of the twelve youth prisons in the German Democratic Republic, known as Jugendhäuser, the one in Halle was notorious for the violence among its inmates. Self-harm in the form of suicides and attempted suicides had been common throughout the prison’s existence since 1971, but hunger strikes also became frequent in the 1980s. The aim of this paper is to explain the causes of self-harm in the Jugendhaus Halle, the risks for young people and the chances of achieving their goals, and to discuss a concept of “nonviolent” resistance in this context.

**Methods:**

We analyzed the personal files of juvenile prisoners from the Archive of the Correctional Facility Halle and the Stasi Records Archive Halle concerning the situation and incidents in the Jugendhaus Halle. The historical-critical method was used to analyze these sources.

**Results:**

Most of the youths involved in the hunger strikes were political prisoners who wanted to leave East Germany and felt their sentences were unjust. The hunger strikes usually lasted one or two days, but in persistent cases, the youths were force-fed. Suicide attempts were caused by abuse and humiliation. After medical treatment, those attempting were restrained in “chain beds.”

**Conclusions:**

While suicide attempts expressed despair, hunger strikes were frequent among political prisoners protesting their sentences and demanding freedom of movement. Self-harm as a resistance was largely ineffective and met with punitive measures. The idea of “nonviolent” resistance through self-harm is controversial, as it involves harming one’s own body and often provokes further violence by prison authorities.

## Introduction

Self-harm is mainly associated with some personality or mental disorders, such as schizophrenia, depression, anxiety, borderline and antisocial personality disorders. As the International Classification of Diseases (11th Edition, ICD-11) does not distinguish between suicidal and non-suicidal self-harm [1], self-harm can be defined as an act where a person “deliberately harms themselves irrespective of the method, intent or severity of any injury” [2]. In the prison context, studies suggest that self-harm is associated with feelings of hopelessness and helplessness, overwhelming guilt, self-punishment, a lack of satisfaction and a desire to escape isolation [3]. However, self-harm can also be a form of protest against violence in prison, sentencing and length of imprisonment. As self-harm in the form of suicide attempts and hunger strikes was particularly common in the 1980 s in the juvenile detention center in Halle, we want to evaluate the reasons for this behavior of young people, their risks and chances of achieving their goals, and discuss a concept of “nonviolent” resistance in this context.

For our investigation, we chose to examine a juvenile detention center in the German Democratic Republic (GDR), a one-party communist state in the Eastern Bloc. The ruling party, the Socialist Unity Party of Germany (SED), declared the superiority of the socialist social order but did not allow its citizens to question its policies or communist ideology. Since criminal acts were considered incompatible with a socialist society, the main causes of crime were seen as bourgeois ideology and the influence of the “class enemy.” Consequently, the GDR criminalized and persecuted the politically motivated behaviour of its citizens, including young people [6].

**Fig. 1:**
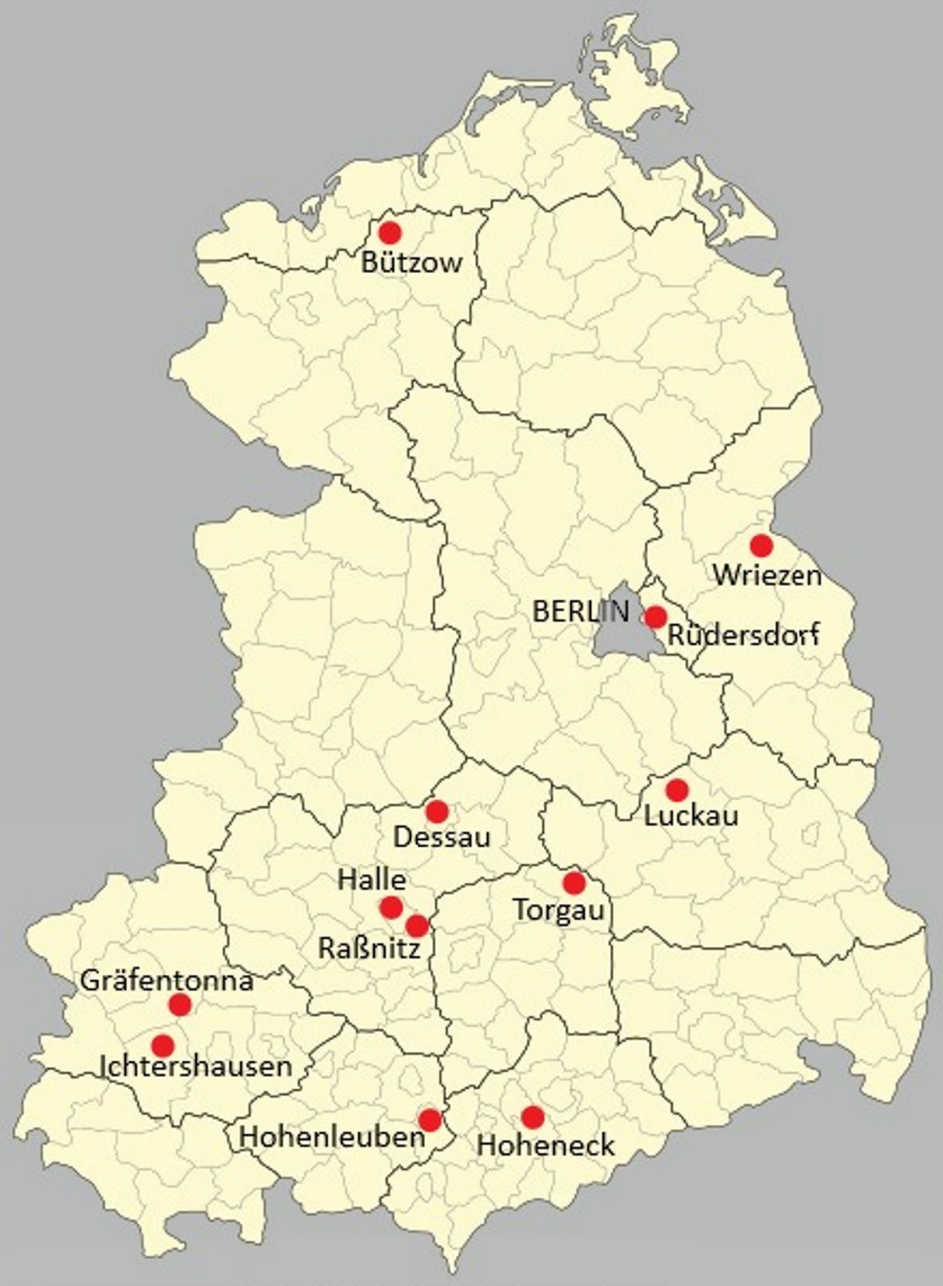
Jugendhäuser in East Germany (Visualization by Oxana Kosenko, based on a map of the districts of the former GDR from 1966, which is in the public domain)

In 1952, youth prisons called *Jugendhäuser* were established in the German Democratic Republic (Fig. 1). Among twelve Jugendhäuser the one in Halle was notorious for the violence that prevailed there. Founded in 1971, it was considered to be one of the most modern penal institutions of its time. Initially it had a capacity of around 1,200 juveniles, but in 1981 its capacity was increased to 1,800 juveniles as a result of reconstruction work. The Jugendhaus Halle housed young people under the age of 18 as well as a number of adult prisoners. According to GDR statistics, in 1981 at least half of the adult prisoners were young people aged between 18 and 25. By September 1982, three quarters of adult prisoners were under 25 [4]. All the prisoners were male. Throughout the 1980 s, many political prisoners were held in the Jugendhaus Halle. They were sentenced under paragraphs 213 (“illegal border crossing”) and 220 (“defamation of the state”) of the GDR penal code [4]. In 1983, the emigration procedure in the GDR was officially regulated for the first time. While between 1973 and 1983 an average of 9,000 people a year left the GDR for the Federal Republic of Germany (FRG), in 1984 41,000 people applied to emigrate [5]. In 1985, for example, 99 inmates of the Jugendhaus Halle applied to leave the country, and in 1986 225 prisoners did so [4].

The aim of the Jugendhäuser was not only to punish adolescents for their crimes, but also to re-educate them into socialist personalities. Educational success was to be achieved through socially useful work, vocational training, civic and general education, cultural and sports activities, the enforcement of order and discipline, and a system of self-education. Following the model of collective education developed by the Soviet pedagogue Anton Makarenko (1888–1939), the prisoners were divided into groups, each of which had its own “educator.” However, the educator delegated some of his authority to selected young people, who often overstepped their authority and used violence against other members of the group. Prisoners at the bottom of the hierarchy were subjected to harassment, psychological and physical torture and sexual assault. Educators often tolerated these violent excesses. The prison system did not separate political and criminal prisoners [4, 6, 7]. The young people with learning disabilities and mental health problems were allocated to the “special section” [4].

The history of Jugendhäuser has recently attracted the attention of historians. However, the research on it is limited to Halle and Dessau. The main themes are political persecution and the history of everyday life (*Alltagsgeschichte*) [4, 6–9]. The medical aspect of detention was a subject of one special investigation [10]. In this paper, we aim to deepen the existing research and explore such an important aspect of detention and a major issue in the Jugendhaus Halle as self-harm, its causes and treatment. We structured our paper as follows: firstly, we touch on the conditions of detention in the Jugendhaus Halle in the 1980s. Secondly, we examine forms of self-harm, such as hunger strikes and suicides or attempted suicides. Then, we compare these results with the experiences of the former young prisoners themselves and evaluate a concept of “nonviolent” resistance in this context.

## Materials and methods

We analyzed the personal files of the former adolescent prisoners of the Jugendhaus Halle, which are stored in the Archive of the Correctional Facility Halle, as well as unpublished documents from the Stasi Records Archive Halle (Stasi-Unterlagen-Archiv Halle), which is part of the German Federal Archives (Bundesarchiv). The Ministry of State Security, commonly known as the Stasi (an abbreviation of “state security” or *Staatssicherheit*), was the secret police, counterintelligence, and intelligence agency of the German Democratic Republic. The Stasi influenced nearly all aspects of life for GDR citizens. It had an extensive network of informants, known as “unofficial collaborators” (*inoffizielle Mitarbeiter*), in the GDR and Western countries. The Stasi infiltrated the Jugendhaus Halle with such informants. They passed relevant information to Department VII of the Halle district administration. This department supervised and instructed the People’s Police and the Ministry of the Interior in the Halle district. These documents are important, because they contain excerpts from reports on incidents at the Jugendhaus Halle, as well as other data that informants provided to Stasi officers. We evaluated 71 archival files from the Stasi district administration in Halle. The files contain reports about the situation in the Jugendhaus Halle, characteristics of the educators and other prison staff, and other related information from “unofficial collaborators” such as “Oliver,” “Falke,” and “Fred.” The documents also contain copies of internal correspondence from the Jugendhaus Halle, as well as internal documentation from Department VII and other Stasi departments. The most detailed data on incidents can be found in the reports from 1982 to 1983 (BArch, MfS BV Halle Abt. VII VIII 1295/69 Bd. 4, 5), which note not only the type of incident, but also the motives of the adolescents and the consequences they faced. A report from 1989 (BArch, MfS BV Halle Abt. VII, 1007) is less detailed, as motives of the adolescents were rarely noted. Internal documentation from Stasi Department VII of the Halle district administration reflects incidents in January and February of 1984 at the Jugendhaus Halle (BArch, MfS BV Halle AOP 22/85), as well as daily observations during the adolescents’ hunger strikes in October of 1989 (BArch, MfS BV Halle Abt. VII, 843).

In the Archive of the Correctional Facility Halle, we have selected psychological assessments of political prisoners. The Archive of the Correctional Facility in Halle holds a total of about 15,000 files of the Jugendhaus Halle from 1972 to 1990. We evaluated about 600 files from different years, or around 300 files from 1980s.

We chose the 1980 s for several reasons. Firstly, it is better documented than earlier years. At least in the case of self-harm, we were able to find the most information. Secondly, the change of political course in the country led to relevant socio-economic changes. In the 1980 s, the Halle Jugendhaus was filled with many political prisoners as citizens of the GDR became increasingly dissatisfied with their living conditions and attempted to move to West Germany, both legally by applying to leave the country and illegally by fleeing across the border. It is therefore important for us to trace whether self-harm was correlated with the political views of the young prisoners. We also chose the Jugendhaus Halle for our paper because the documentation of self-harm there is better documented than that of the Jugendhaus Dessau, which was also notorious for the violence that occurred there [6, 10].

In order to examine these sources, we used the historical-critical method, which includes the stages of acquiring primary sources and research, critically evaluating the information contained in the primary sources, and presenting historical data in its historical context in terms of objectivity and significance [11].

## Limitations

The most complete information on self-harm among former adolescent prisoners in the 1980 s is available for the years 1982, 1983, partially for 1984, and 1989. For the remaining years, only fragmentary information is available. Since the original reports from Jugendhaus Halle are not preserved, we rely on the information contained in the documents from the Stasi Records Archive in Halle. It is well known that, on the eve of profound political changes in the GDR in the fall of 1989, the Stasi actively destroyed its documentation, which explains the gaps in its records [12]. Information about incidents at the Jugendhaus Halle was relayed to the Stasi through its informants. Although it was not an official statistic, it can be considered accurate, because the Stasi had multiple informants and could compare all the information received. However, certain biases cannot be excluded because it was not an official source. Despite the gaps in archival documentation, which are a common issue for historians, the files provide insight into cases of self-harm during this period at the Jugendhaus Halle.

## Results

### Detention conditions in Jugendhaus Halle in the 1980s

Before proceeding to a description of self-harm cases in Jugendhaus Halle, it is necessary to outline the conditions of their detention in general. Jugendhaus Halle was a notorious place for juvenile prisoners because of the violence that prevailed there from the time it was established in 1971. This situation remained unchanged throughout the 1980s. As officials of the Stasi noted in 1983, the unrestrained brutality, maltreatment, including sexual abuse, harassment, humiliation and beatings among juveniles were typical of the Jugendhaus Halle [13]. The violent and humiliating acts were committed over a long period of time without being fully investigated by the authorities of the Jugendhaus. The incidents in the groups of adolescents were only superficially investigated by the responsible educators. This is not surprising given that most of them was not interested in doing their jobs. Among the characteristics on nine educators in 1984, only two were positive. Most of the educators were not able to help adolescents with their problems, had little knowledge of their work, and spent a lot of time in their offices drinking coffee [14].

In 1983, there were a total of 149 incidents of beatings and other violence by detainees against other detainees, including several assaults on prison staff. There were also 30 suicides or attempted suicides [15], Table 1. Despite four criminal proceedings against twelve juveniles in 1983, two serious incidents occurred in 1984 [13]. Some incidents were not recorded or not clarified to the end. However, unofficial collaborators of the Ministry of State Security in the Jugendhaus Halle informed about the situation and incidents. Thus, this information reached the Ministry of the Interior, which sent a control group to investigate and eliminate those conditions to the Jugendhaus Halle, which ended with the removal of the head of the Jugendhaus and the appointment of a new one [16]. However, the situation did not improve and even worsened due to political prisoners who regularly engaged in passive resistance, as will be discussed in detail in the following sections.

### Nonviolent resistance and hunger strikes

If we talk about aggressive forms of resistance in the Jugendhaus Halle, such actions happened there: insulting or beating prison staff, escaping, causing machine damage in workshops, writing protest slogans on walls, tables, and other objects (damage to property/graffiti) [17]. However, adolescents preferred nonviolent resistance, such as hunger strikes, suicidal attempts or suicides, refusal to work.

Hunger strikes were a form of protest primarily practiced by political prisoners (Table 1). It is important to acknowledge that the Jugendhaus staff stigmatized political prisoners. Unlike previous years, personal files from the 1980 s include records of intake interviews. In these files, prison psychologists described the basic characteristics of delinquent adolescents and predicted how they would behave among fellow inmates. For example, when describing J.A.’s personality, a 17-year-old boy imprisoned for illegally crossing the border, the psychologist noted that he had no friends and was often seen “loafing at work.” Due to “considerable educational difficulties,” J.A. spent two years in a *Jugendwerkhof*, or “youth work yard”—a special home for juveniles considered difficult to educate. Regarding his crime, J.A. said that life would be better in the Federal Republic of Germany and that he wanted to go there and apply for resettlement. Despite noting that J.A. was quiet and reserved, had no friends, and was “mentally retarded” and “primitive in his thoughts and actions,” the psychologist assumed that the boy would come into contact with negatively inclined young people and violate norms. The psychologist also expected J.A. to spread Western ideology “in his primitive way” [18]. However, as his time at the Jugendhaus showed, this prediction was incorrect: J.A. behaved calmly and was well-behaved [19].

We do not have exact statistics on hunger strikes for the entire 1980 s, but, for example, there were at least 30 hunger strikes in 1982 (Table 1). Once it was established that the adolescent had missed a meal intentionally, he was immediately separated from the other cellmates in the group, after which after which have they been asked to explain their behavior. Political prisoners reasoned that their refusal to eat was because they wanted their applications to leave the country to be processed more quickly. Others wanted to force a review of the verdict because they considered it to be unjustified [20]. There were also some personal reasons. One juvenile prisoner explained that he could no longer withstand, because he was constantly abused by other prisoners. Another juvenile wanted a transfer to another prison [20].

Usually, refusal to eat lasted one or two days. Typically, after talking to prison officers, the juveniles would end their hunger strikes. Apparently, for adolescents hunger strikes were a way of communicating their aspirations to their superiors in a direct way. As there was usually no solution to their problems, they went on hunger strike several times. Usually, hunger strikes were combined with a refusal to work. In cases where adolescents stubbornly refused to eat, they were either force-fed in the Jugendhaus or admitted to the prison hospital [20].

On October 5, 1989, 213 of the 265 political prisoners convicted under § 213 (“illegal border crossing”) of the GDR penal code began a hunger strike and refused to work. They heard that many GDR citizens who had sought refuge in the West German embassy in Czechoslovakia were able to leave for West Germany. When thousands of their fellow citizens were officially allowed to leave the GDR, the prisoners sentenced under § 213 became even more aware of the absurdity of their situation. With their act of protest, the young prisoners wanted to ensure that their applications to leave the GDR would be considered by the state authorities and that their problem would be solved within the legally stipulated time [21]. Some of the hunger strikers fasted for more than four days, followed by a medical examination. The leaders of the protest fell into this category. It was not until October 10 that all prisoners returned to regular meals [22].

In November 1989, hunger strikes resumed among young prisoners sentenced under § 213. The resumption of the hunger strikes was triggered by further political developments after the fall of the Berlin Wall and the so-called “Monday demonstrations” against the GDR government, where people were already demanding German reunification. The young prisoners cited two main reasons for their refusal to eat and work: their belief that they were unjustly imprisoned and the beatings they had suffered at the hands of prison guards. The youths also wrote letters to the General Secretary of the Socialist Unity Party of Germany, the Chief Prosecutor, and the Supreme Court of the GDR, requesting that their cases be reviewed [23].

It should be noted that other forms of passive protest have also led to reprisals by the authorities of the Jugendhaus. For example, in 1989, when a prisoner refused to do cleaning work, a chain twister (*Führungskette*) was placed on him [17].

### Suicides and suicide attempts

Attempted suicides were common in the Jugendhaus Halle. In 1983 alone, at least 30 suicide attempts were recorded [15]. Most of the suicide attempts were made by opening veins with razor blades. Sometimes there were cases of hanging with a shirt sleeve or ripped bed sheets [17] or attempted suicide by swallowing objects. For example, in 1989 a prisoner swallowed parts of a broken porcelain sink in the toilet [17]. In the 1980 s, the suicide attempt was usually followed by a visit to a psychologist and neurologist [20]. However, there are no psychiatric or neurological reports in the inmates’ personnel files.

The reasons for the suicide attempts were mostly caused by the conditions of detention in the Jugendhaus, such as physical bullying, beatings and moral humiliation. For example, one young prisoner cited sexual abuse by his groupmates as the reason for his suicide. Using a razor blade, he inflicted a cutting wound approximately 5 cm long and 3 mm deep on his left wrist [20] (Tables 1 and 1982). There were also some cases where the suicide attempt was a protest against the sentence. The inmates were in such desperate circumstances that they threatened prison staff with breaking the house rules or even committing suicide (Table 1). There have also been cases where a juvenile prisoner even helped his cellmate to commit suicide. For example, in 1982, an adolescent attempted suicide by cutting a 15-cm-long wound on his left wrist with a razor blade. His cellmate then helped him make a 10-cm cut on his right wrist. The juvenile who attempted suicide was serving a sentence for theft and attempted escape from the GDR. He also received an additional two years for “prisoner mutiny.” The incident was to be investigated by the responsible educator. Like all juveniles who attempted suicide, he was isolated. His wounds were covered with bandages. His hands were tied to the “chain bed” [20]. The “chain bed” had hand and ankle cuffs attached to all ends of the bed so that the prisoner could be locked in with his arms and legs spread and could hardly move. Indeed, in many cases not only the hands, but also the feet of adolescents who attempted suicide were tied to the bed in order to “protect their own person” [24]. Even in cases where a youth expressed suicidal intentions, he was given hand and foot restraints and separated from the group. After the restraints were removed, he was checked every half hour [17].

Despite repressive measures being put in place to prevent suicide and first aid being provided in a timely manner, there were still rare cases of adolescents successfully taking their own lives. One such case occurred in 1980, when a 15 year-old pupil, hanged himself with his shoelaces in the Jugendhaus Halle. The boy had been sentenced to twelve months’ imprisonment for “illegal border crossing.” According to prison staff, he had previously been bullied by other young people “due to his lack of social contacts and excessive sensitivity.” This bullying continued in prison, but did not lead to any physical altercations. Mehlis, who “suffered from severe depressive moods, particularly after being bullied, apparently committed suicide as a result of his mental depression” [25].

## Discussion

### “Nonviolent” vs. violent resistance

Traditionally, the forms of protest in the prison environment are conditionally divided into nonviolent (passive) and violent (active) resistance. Nonviolent resistance, or nonviolent action, is the practice of achieving goals through disobedience, while refraining from violence or the threat of violence. Active or aggressive forms of protest include actions by inmates directed at other people and their environment that may result in violations of the law or prison order, as well as harm to others or damage to property.

Hunger strikes are also considered to be a form of nonviolent protest that occurs mostly in the prison environment, where prisoners or detainees aim to “to obtain certain goals by inflicting negative publicity on the authorities” [26]. There are special research works on hunger strikes written by political scientists [27, 28]. However, by describing bodies as “political structures”, researchers make these bodies appear abstract. Since hunger strikes can cause severe health complications [27], this form of resistance can be seen as violence against one’s own body. Under current medical practice, a hunger striker must be medically examined within 24 h and a doctor must explain the health risks of fasting to the striker. A daily follow-up must follow to assess the striker’s health and desire to continue fasting [29]. In the Jugendhaus Halle a medical examination usually followed after four days of fasting. The strikers were not informed of the risks of fasting, but of the criminal consequences [4]. According to the recollections of a prisoner who participated in the hunger strike in October 1989, he and another prisoner were shackled to the “chain bed” as were those who attempted suicide [4]. Others were handcuffed to the bars of the ward for several hours so that they could only stand on their toes, which was extremely uncomfortable and painful [4]. One hunger striker recalled that he and other prisoners drank water and tea during the strike, apparently to avoid serious health consequences from fasting [4]. It is unclear whether they were informed of this by the prison staff. Another young prisoner recalled that he and two other young people had been on hunger strike for 10 days, but they went to the doctor every day to have their blood pressure measured and to weigh themselves. After a week, the head of the Jugendhaus said there was no way they could leave the GDR and threatened to force-feed them. At the same time, he accepted the request of one of the strikers to be transferred to another group of inmates. Some of the strikers were indeed tied to a bed and force-fed [4]. Although all the prisoners ended their hunger strike on October 10, 1989, 29 prisoners still refused to work. They were punished with three weeks’ arrest and one was even investigated for “prisoner mutiny” [4]. As in other countries, when the prison authorities failed to recognize a strike or refused to negotiate, they separated strikers or strike leaders and took punitive measures [30].

As for suicide attempts, like hunger strikes, they could be seen as a non-violent form of protest against the environment and prison conditions, but violent against the health of the suicidal person themselves. The cases mentioned above show that suicide attempts were punished by prison staff. Tying juveniles to the “chain bed” can be called torture in order to prevent them from attempting suicide again. Instead of helping the suicidal person to solve his problems, which was often within the power of the authorities of the Jugendhaus, they only received punishment. The case of a prisoner who refused to work, after which chain twister was put on him, is also illustrative of this.

It can be concluded that self-harm by juvenile prisoners was not only ineffective in achieving their demands, but in most cases was punished with “chain beds,” force-feeding, and arrest. In 1975, the World Medical Association declared that doctors should not participate in the force-feeding of hunger strikers because it was considered inhumane and ethically unacceptable. Since then, this requirement has been renewed several times, most recently in 2017 [28]. As we have seen, the declaration of the World Medical Association was ignored in the GDR.

Thus, from a medical point of view, the concept of “nonviolent” resistance or protest in the form of self-harm seems controversial, as it implies violence to the bodies of those who protest. Moreover, this kind of protest turned out to be violence against them by the prison administration. As we can see in this case, the punitive reaction of the prison authorities to the hunger strike has common features in those countries where the rights and freedoms of the citizens have been violated.

### Reasons for self-harm

If we examine the reasons for hunger strikes, we can see that this form of protest as such appears mainly among political prisoners in the 1980 s and is also largely political in nature, namely advocating the possibility of emigrating to the FRG and disagreeing with the verdict. There is no information about hunger strikes in the 1970s. It is generally believed that the tradition of hunger strikes as a form of nonviolent protest has its origins in political struggle [31]. We can clearly see that the hunger strikes at the Jugendhaus in 1989 began as a response to major political events in the country, such as antigovernment demonstrations. While the hunger strikes were primarily a protest against imprisonment per se and an attempt to leave the GDR, the suicide attempts were more an expression of desperation and a response and protest against physical and moral violence in the Jugendhaus.

Despite the high number of attempted suicides in the Jugendhaus, prevention was effective due to constant supervision and punitive measures against those who attempted suicide or expressed intentions to kill themselves. In the GDR, prison staff were even trained in suicide prevention, so that the suicide rate in GDR prisons was lower than in West Germany [10]. It is interesting to note that in the 1970 s, the most common method of suicide in the Jugendhaus was the swallowing of foreign objects, such as screws or tablespoons, which prisoners might have stolen from the workshop or kitchen [10]. In the 1980 s, wrist-cutting suicides were preferred. It seems that in the 1970 s it was forbidden to have objects in prison that could be used to injure oneself.

The association between violence and self-harm, in particularly subsequent suicide attempts, has been well researched and documented. Psychiatrists have found that sexual, interpersonal, and perpetrator violence are more likely to lead a victim to attempt suicide than other forms of trauma [32]. Research on adolescents has shown that exposure to violence is more strongly associated with suicidal attempts and non-suicidal injury than exposure to non-violent traumatic experiences [33]. Repeated exposure to violence is an especially strong risk factor for suicide [34]. When discussing the intentions and motives of individuals exhibiting suicidal behavior, psychiatrists distinguish between “suicide attempters”- individuals who “made a serious attempt to kill themselves and it was only luck they did not succeed,” individuals who “made a serious attempt to kill themselves, but knew their method was not foolproof,” and “suicide gesturers”- individuals who made a suicide attempt, but did not intend to die and were instead seeking help [34]. Since we are dealing with retrospective data, it is difficult to evaluate whether the suicidal attempts of adolescent prisoners in the Jugendhaus Halle were made with a strong intent to die or if they were rather “suicide gestures.” Considering the dangerousness of the self-harm methods used (e.g., long and deep cuts to the wrists or attempts at hanging), we could suggest that the adolescents made serious attempts to kill themselves. Moreover, given the frequency of suicide attempts at Jugendhaus, the young inmates must have been aware of the potential punishment and the likelihood that their situation would not improve after a failed attempt. As already Sykes pointed out, prisoners “will revolt in a desperate situation even though they know there is no chance of success” [35]. Therefore, we assume their motivation stemmed from hopelessness about their situation and an unwillingness to obey prison and justice system rules. We can also assume that adolescents who wanted to improve their situation without risking their lives chose a hunger strike as a form of protest.

## Conclusion

Self-harm in the Jugendhaus Halle was a form of protest against prison conditions or even against state authorities. Suicide attempts were an expression of despair and a response to physical and moral violence. Hunger strikes were more common among political prisoners who were protesting their sentences and demanding their right to freedom of movement. In general, self-harm by juvenile prisoners was not only ineffective in achieving their demands, but in most cases was punished with “chain beds,” force-feeding, and arrest. In the GDR, the autonomy of hunger strikers was not respected according to the Declaration of the World Medical Association, so force-feeding took place. Both shackling to “chain beds” and artificial force-feeding are now considered torture. Thus, the concept of “nonviolent” resistance in the form of self-harm seems controversial, as it implies harm to one’s own body and often physical violence or even torture by the prison administration.

**Table 1:** Suicides, suicide attempts and hunger strikes occurred at the Jugendhaus Halle in 1982–1984, and 1989

Year	Number of suicides and suicide attempts, and motives	Number of hunger strikes and motives
1982	1 suicide. A total of at least 12 suicide attempts, where: 7 cases—physical bullying, beatings, sexual assault and moral humiliation by other inmates (2—“difficulties” with inmates in the group; 2—“difficulties” with other inmates; 1—abuse and bullying by other inmates; 1—beatings by other inmates; 1- sexual assault); 1 case—fear of being beaten up by other inmates because of a 60-mark debt; 1 case—unresolved personal problems, including being banned from watching television due to violating fire safety regulations; 2 cases—protest against an “unfair” or “harsh” judgment; 1 case—“does not want to live” (the adolescent was imprisoned for theft, attempted escape from the republic, and an additional two years for prisoner mutiny). One adolescent threatened to commit suicide because he felt threatened by the other inmates.	A total of 30 cases, where: 6 cases—disagreement with the judgment; 4 cases—insistence on processing their applications to leave for the Federal Republic of Germany; 3 cases—disagreement with disciplinary punishment; 2 cases—insufficient medical treatment; 2 cases—disagreement with transferring to another jurisdiction area of the Jugendhaus; 2 cases—insistence on transferring to another correctional facility; 1 case—moral humiliation from inmates; 10 cases—no data.
1983	A total of 30 suicide attempts, where: 2 cases—physical bullying, beatings and moral humiliation; 2 cases—disagreement with the judgment; 16 cases—no data. Three adolescents threatened to commit suicide: 1—due to insufficient ophthalmological care; 2—no data.	No data.
1984	4 suicide attempts during January and February, where: 4 cases—physical bullying, beatings and moral humiliation.	No data.
1989	A total of at least 5 suicide attempts, where: 1 case—physical bullying, beatings and moral humiliation; 4 cases—no data. Eight adolescents threatened to commit suicide (no data about motives).	At least 201 cases, where: 215—insistence on processing their applications to leave for the Federal Republic of Germany (213 cases in October 1989, the number of hunger strikers in November 1989 is unknown). 1 case—disagreement with the judgment.

Source: References 14–23.

## Data Availability

No datasets were generated or analysed during the current study.
